# A Framework for Assessing the Permissibility of Academic Leaders’ Outside Activities

**DOI:** 10.1111/1468-0009.70024

**Published:** 2025-06-04

**Authors:** MATTHEW S. McCOY, MARTHA E. GAINES, STEVEN JOFFE, GENEVIEVE P. KANTER, EMILY A. LARGENT, BERNARD LO, HOLLY FERNANDEZ LYNCH, ALLISON M. WHELAN, MICHELLE M. MELLO

**Affiliations:** ^1^ Perelman School of Medicine, University of Pennsylvania; ^2^ Leonard Davis Institute of Health Economics, University of Pennsylvania; ^3^ University of Wisconsin Law School, University of Wisconsin–Madison; ^4^ Sol Price School of Public Policy, University of Southern California; ^5^ University of California San Francisco; ^6^ The Greenwall Foundation; ^7^ College of Law, Georgia State University; ^8^ Stanford Law School; ^9^ Stanford University School of Medicine; ^10^ Freeman Spogli Institute for International Studies Sanford University

**Keywords:** ethics, conflict of interest, academic medical centers, universities, governance

## Abstract

**Policy Points**

- Many have urged academic institutions to rethink conflict of interest policies governing leaders’ outside activities, which pose not only individual conflicts for leaders themselves but institutional conflicts for their academic employers.
- Although the American Association of Medical Colleges and Association of American Universities have provided guidance on managing such conflicts, neither offer a structured approach for determining when and under what conditions it is appropriate for a leader to engage in specific outside activities.
- To address this gap, this article develops a decision‐making framework that institutional oversight bodies can use to assess the permissibility of academic leaders’ proposed outside activities.

Over the past several years, investigative reports from the new York Times, ProPublica, The Boston Globe, and other media outlets have renewed public scrutiny of the practice of academic leaders, such as medical school deans and health system chief executive officers (CEOs) serving on the boards of for‐profit health care companies.^1^, ^2^, ^3^, ^4^, ^5^, ^6^ Building on prior academic studies,^7^, ^8^, ^9^ the recent wave of reporting revealed that leaders at many of the country's top academic medical centers earned—in addition to their institutional salaries—hundreds of thousands of dollars a year as directors of pharmaceutical and device companies. In the wake of these reports, critics have urged academic institutions to rethink conflict of interest policies governing leaders’ outside activities, which are understood to pose not only individual conflicts for leaders themselves but also institutional conflicts for their academic employers.^10^, ^11^, ^12^

Institutional conflicts of interest (ICOI) “arise when an institution's own financial interests or those of its senior officials pose risks of undue influence on decisions involving the institution's primary interests.”^13^ The reason academic leaders’ individual financial relationships are seen as having the potential to generate ICOI is that these individuals’ administrative decisions shape institutional policy and processes, often with far‐reaching consequences. When an academic leader assumes a board position, it raises the concern that financial incentives and fiduciary duties linked to the outside role may influence the leader to act “in ways that favor the interests of the company but compromise the overall research, educational, or clinical mission of the institution.”^13^ Leaders’ outside roles can also create “conflicts of commitment,” which arise when spending time and effort on outside activities interferes with leaders’ ability to carry out their institutional responsibilities.^13^

Despite calls for reform, limited guidance exists for academic institutions seeking to strengthen ICOI policies. A 2008 report by the Association of American Medical Colleges (AAMC) and the Association of American Universities (AAU) details strategies for managing ICOI arising from leaders’ external activities, and a 2009 report from the National Academy of Medicine (NAM) outlines best practices for establishing an ICOI committee.^13^, ^14^ However, neither of the reports provides a structured approach for determining when and under what conditions it is appropriate for a leader to pursue a particular outside activity. It is unsurprising, then, that responses to the recent critical media coverage reflect continuing uncertainty about the potential risks and benefits of academic leaders’ involvement with outside entities and how those factors should be weighed in specific cases. Whereas some academic institutions have significantly curtailed leaders’ interactions with companies, others maintain permissive policies.^15^ The lack of clear standards in this area is concerning because it could result in institutions maintaining or adopting poorly designed ICOI policies and procedures that either underregulate or overregulate leaders’ outside activities.

To address these challenges, we provide a decision‐making framework that institutional oversight bodies can use to assess the permissibility of academic leaders’ outside activities. To facilitate use of the framework, we also develop a taxonomy of potential benefits, risks, and management strategies relevant to academic leaders’ involvement with outside entities. Finally, we discuss application of the framework.

Although the framework prescribes standardized decision steps, it is flexible enough to guide deliberations across cases involving different leadership roles (e.g., department chairs, deans, provosts, health system CEOs, presidents) and external activities (e.g., consulting, board service) and to allow for reasonable variation in decision making across institutions. Additionally, although our argument is motivated by and draws on examples from recent debates about ICOI within academic medical centers, the framework is applicable to other academic contexts and, possibly with minor modifications, to other institutions that provide patient care or conduct human subjects research.

## Decision‐Making Framework

Our framework builds on foundational work by Pisano and colleagues,^16^ who introduce a “compelling institutional interest” standard for assessing the permissibility of academic leaders’ outside activities. According to that standard,

…the fiduciary responsibilities of presidents, provosts, vice presidents, deans, chief executive officers, and those who report to them should preclude a paid relationship with an outside entity in related and relevant areas unless a case can be made that there is a compelling institutional interest in the leader's service in such a role.^16^

Before turning to the ways in which our framework modifies and extends the compelling institutional interest standard, we begin by addressing two important qualifications in Pisano and colleagues’ formulation of the standard: namely, that it should be applied to *paid* relationships with entities in areas *related and relevant* to those of the academic institution.

Pisano and colleagues’ emphasis on paid relationships implies that such relationships ought to be treated in a fundamentally different manner than unpaid relationships. We reject this dichotomy. It is true that paid relationships involve financial incentives that may increase the risk of biased decision making or the likelihood that such relationships will be seen as inappropriate. Oversight bodies should take these aspects of paid relationships into account when assessing them. However, unpaid relationships also may carry risks that oversight bodies should review carefully. For example, a medical school dean could decline compensation for service on a pharmaceutical company's board of directors. Although unpaid, such a relationship entails competing fiduciary duties and a substantial time commitment that might interfere with the dean's ability to carry out primary responsibilities. Rather than applying categorically different levels of scrutiny to paid and unpaid relationships, then, oversight bodies should carefully assess specific risks presented by a proposed activity.

We agree with Pisano and colleagues that a primary consideration for oversight bodies should be whether a proposed activity involves an outside entity with interests in areas related and relevant to the mission of the academic institution. For example, if an academic medical center's CEO joins the board of a medical device company, the activity would involve an entity with interests clearly related to those of the academic institution, whereas a similar role on the board of a symphony would not. The distinction is ethically relevant because the former case presents greater risks to the academic institution. Because many of the CEO's administrative decisions could implicate the interests of the device company, there is a danger that those interests will influence the CEO's decision making at the expense of the institution. By contrast, there are few imaginable scenarios in which the CEO's decision making for the institution would implicate the interests of the symphony.

In practice, whether an outside entity's interests are related to those of the academic institution will typically be a question of degree. Rather than asking whether an outside entity's interests are or are not related to those of the academic institution, oversight bodies should ask how closely they are related. When the outside entity's interests are only marginally related or unrelated, we share Pisano and colleagues’ view that the activity should be subjected to a lower standard of scrutiny. Provided the activity involves a reasonable time commitment and does not present reputational risks, it should generally be permitted.

Where an external role does involve an entity with interests in areas that are closely related to those of the academic institution, Pisano and colleagues’ standard directs oversight bodies to ask whether the activity serves a compelling institutional interest. This requirement is based on the principle that academic leaders’ primary obligation should be to their academic employers and, thus, that there should be a presumption against accepting an external role that could jeopardize their ability to carry out these obligations. This principle is well founded. Those with leadership positions at academic institutions freely accept their roles and the responsibilities they entail. They are also typically well compensated and enjoy high levels of esteem.^17^, ^18^ There is no reason to believe that they should be permitted to pursue outside work that exposes their institutions to risk unless their doing so substantially advances the mission of the institution.

Building on these insights, we propose several revisions and extensions to the compelling institutional interest standard. Our aim is to develop a more explicit decision‐making framework that highlights additional ethical considerations relevant to assessing the permissibility of academic leaders’ outside activities.

First, although the concept of a compelling interest test has an established place in American constitutional law, it may be challenging to apply in this context because it requires an oversight body to determine which of the institution's many interests count as compelling and potentially to weigh countervailing interests against each other. Instead, we suggest that the oversight body should initially ask whether the activity is reasonably likely to yield substantial institutional or societal benefits. We include the qualifier “reasonably likely” to acknowledge that it is difficult to know at the outset whether an external relationship will yield anticipated benefits. However, as discussed in the next section, ongoing review of external activities should assess whether they are in fact proving beneficial. Additionally, whereas Pisano and colleagues argue that the outside activity must be justified by a compelling *institutional* interest, we argue that it might also be justified by its societal benefits, even if it is not likely to benefit the institution directly. For example, an academic leader's involvement with a government advisory committee or a professional society may do little to benefit the institution but could produce societal benefits by enhancing the work of those bodies. This expansion of the institutional interest standard to include societal benefit is consistent with the broader missions of academic institutions.^19^

Second, we argue that a reasonable likelihood of producing institutional or societal benefit is necessary but not sufficient to justify an outside activity. If an outside activity is deemed likely to be beneficial, the oversight body should next ask whether the outside activity presents ICOI or related risks to the institution. This requirement is grounded in a basic consequentialist principle, which is that to be justifiable, the expected benefits of the external activity must outweigh its expected risks to the institution. An implication of this requirement is that not all potentially beneficial external relationships, even those likely to yield substantial institutional or societal benefits, should be permitted.

Third, when an outside activity does present conflict of interest or related risks, the oversight body should ask whether those risks can be managed at a reasonable cost to the institution, where “reasonable” should be understood in terms of proportionality to the benefits expected from the outside activity. An extension of the consequentialist principle noted previously, this requirement is intended to account for the fact that many strategies for managing ICOI impose administrative costs on the institution. For example, part of the Dana‐Farber Cancer Institute's approach to managing conflicts of interest stemming from its CEO's directorship of GlaxoSmithKline was to prevent her from overseeing or discussing ten clinical trials and 14 research and licensing agreements with the company.^4^ Although this type of wide‐ranging recusal may mitigate the risk of biased decision making, it carries potentially significant administrative costs because it requires reassignment of a leader's responsibilities to others across the institution. Because these costs are borne by the institution, they must be factored into the decision to permit or prohibit an outside role.

**Figure 1:**
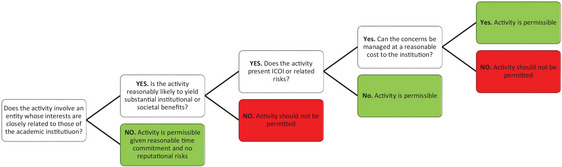
Decision‐Making Framework [Colour figure can be viewed at wileyonlinelibrary.com] ICOI, institutional conflicts of interest.

These revisions and extensions to the compelling institutional interest standard produce a decision‐making framework comprising a sequence of questions for assessing the permissibility of leaders’ outside activities (Figure 1). Although the framework captures relevant ethical considerations in general terms, applying it to specific cases requires careful deliberation. To inform this deliberation and facilitate use of the framework, we provide a taxonomy of potential benefits and risks associated with leaders’ outside activities. We then review common strategies for managing ICOI arising from leaders’ outside activities and assess their applicability to different risk types.

## Benefits, Risks, and Management Strategies

### Benefits

When a proposed activity is determined to involve an entity whose interests are closely related to those of the academic institution (step 1 of the framework), step 2 directs oversight bodies to assess whether the activity is reasonably likely to yield substantial institutional or societal benefit. This task is made challenging by the fact that there has been limited empirical research on the effects of leaders’ outside engagement. Bearing in mind that much of the support for the benefits of outside activities is speculative and anecdotal, oversight bodies should ask the leader to submit a written proposal identifying specific benefits that the activity is expected to produce. With a proposal in hand, the oversight body can assess, to the extent possible, the likelihood that the benefits will be realized if the leader is permitted to pursue the outside activity and, if they are realized, their magnitude. Although each case will present unique factors, and different oversight bodies may reasonably come to different judgments about the magnitude of expected benefits, several broad categories of benefits have been associated with academic leaders’ external engagements and, thus, might warrant consideration.

First, there are a range of ways in which leaders’ external activities might yield institutional benefits. Those who defend the practice of academic leaders serving on corporate boards often argue that these positions allow leaders to gain *insight into industry trends and developments* that can inform strategic decision making or illuminate new avenues of research within the institution.^20^ For example, the chair of Mass General Brigham's board of trustees argued that outside directorships allow academic leaders to see how companies operate in ways that enhance leaders’ ability to do their jobs.^4^ Others argue that leaders’ relationships with companies can promote beneficial *academic‐industry collaborations*, including sponsored research agreements and other partnerships that can boost institutional revenues and research outputs.^21^ It is also possible that leaders’ engagement with external companies could help them cultivate *relationships with potential donors* to the academic institution.

Second, academic leaders’ involvement with guideline committees, medical professional societies, or government advisory bodies might produce broad societal benefits by enhancing the output of these groups. Some might argue that leaders’ engagement with commercial entities may also produce societal benefits insofar as it contributes to the development of products and services tailored to public needs. The head of Dana‐Farber's board of trustees defended the hospital CEO's board service with GlaxoSmithKline in these terms, arguing that the role allowed her to “represent and advocate for the interests of patients” to the pharmaceutical company.^4^ However, oversight bodies should approach this rationale cautiously. It is incorrect to assume that any novel medication or device will benefit society because new products may be ineffective, unsafe, or inaccessibly priced.^22^ Moreover, even in cases where a new product offers significant benefits, it remains a private good for which the company will be compensated through market pricing. Furthermore, it is difficult to argue that the presence of a particular academic leader on a corporate board is essential to society realizing the product's benefits. For example, we are not aware of evidence that it has influenced pharmaceutical companies’ pricing decisions.

Pisano and colleagues^16^ call attention to a specific subset of cases in which a leader joins the board of a company formed to develop intellectual property owned by the academic institution. Perhaps, in such cases, a stronger argument could be made that the leader's role with the company is consonant with the academic institution's broader social mission of responsibly developing and transferring technology. Such an arrangement might also benefit the institution by increasing the likelihood of a positive financial return on its intellectual property.^23^

Third, some have suggested that allowing academic leaders to pursue outside activities benefits academic institutions by enabling them to recruit and retain high‐level talent. This argument assumes that qualified leaders will be disinclined to accept a position at an academic institution that prevents them from working with outside entities, especially compensated work. For example, the Globe's reporting showed that Mass General Brigham rescinded a policy that prohibited top executives from receiving stock for corporate board service because the policy was seen as more restrictive than those of peer institutions and hospital leaders “were demanding more flexibility.”^4^ Notably, however, the reporting also showed that most elite academic medical center CEOs do not hold positions on the boards of publicly traded companies. Thus, there remains uncertainty about the extent to which competitive candidates would be disincentivized from accepting leadership positions at academic institutions that restrict outside activities.

This rationale for allowing leaders to pursue outside roles also rests on dubious ethical foundations. If outside engagement cannot be justified based on the institutional or societal benefits it yields, then academic leaders’ implicit or explicit demands for permissive policies are self‐serving. If academic institutions struggle to attract and retain high‐end talent, they should consider other incentives, including increasing executive compensation for the leaders’ primary functions, before allowing leaders to pursue outside work that creates institutional risks. Of course, were all health systems to adopt standardized policies restricting external compensation, competitive pressure would ease.

After identifying specific benefits that an outside role is expected to yield, oversight bodies should consider whether there are alternative means of securing those benefits that raise fewer or lower risks. Specifically, the oversight body should ask whether it is possible to achieve the same benefit at a lower level of involvement with the outside entity (e.g., serving on a scientific advisory board rather than a board of directors, consulting rather than serving on a scientific advisory board) and/or with the involvement of a less senior member of the academic institution who, given less authority over institutional decisions, poses a lower risk of ICOI. Oversight bodies should require modifications to an outside role if it is possible to capture expected benefits at a lower cost to the institution.

As described previously, before approving an outside activity, oversight bodies should request that leaders submit and have a face‐to‐face meeting to discuss a written proposal of expected benefits. Using these stated expectations as a rubric, leaders should be required to provide periodic updates on benefits being generated by their outside engagement. This periodic monitoring is particularly important given the lack of a robust evidence base supporting assumptions about the positive effects of leaders’ external engagements. Outside activities that are not delivering expected benefits should be discontinued. Standards for how frequently leaders should report back to oversight bodies and what constitutes sufficient evidence of benefit may vary across institutions and types of outside activities, but they should be clearly established and communicated to leaders in advance. Additionally, oversight bodies should require that nondisclosure agreements or other contractual obligations imposed by external organizations do not restrict academic leaders’ ability to describe the benefits of the relationship.

### Risks

It is widely accepted that academic leaders’ outside activities can generate ICOI, but that term captures several discrete concerns that are not always clearly distinguished. Step 3 of the decision‐making framework directs oversight bodies to delineate and consider specific risks posed by a given outside activity. Delineating risks is critical to assessing the extent to which available management strategies can mitigate those risks. Here we list and briefly describe several categories of risk associated with leaders’ outside activities.

First, *financial conflicts of interest* occur when a leader's outside activities introduce financial incentives that risk influencing the leader's professional judgment with respect to primary responsibilities. Although existing commentary and guidance on ICOI tends to focus on ways in which leaders’ outside financial interests could compromise research integrity, biased professional judgment can, depending on the leader's role, affect any component of an academic institution's mission.

Second, *conflicting fiduciary duties* occur when a leader has fiduciary duties to an outside entity, such as the company on whose board of directors he or she sits, that may come into conflict with the leader's duties to the academic institution. Similar to financial conflicts of interest, conflicting fiduciary duties are concerning because they pose a risk that a leader's decisions will be influenced by what is best for the outside entity rather than guided solely by the interests of the academic institution. Indeed, such risks may be more acute in the case of competing fiduciary duties. Unlike financial incentives, which may or may not influence a leader's behavior, fiduciary duties impose legal obligations to act in the interest of the outside entity. Crucially, competing fiduciary duties can exist even if leader declines compensation for board service.

Third, *conflicts of commitment* arise when spending time on outside activities interferes with a leader's ability to carry out his or her institutional responsibilities. Directorships, for example, can be extremely time consuming. The Globe's reporting showed that in 2019, the former president of Brigham and Women's Hospital attended 30 to 40 meetings related to outside board service, amounting to roughly 18 days’ worth of work—even before accounting for the likely substantial amount of time spent traveling to and preparing for meetings.^4^ According to some estimates, time demands on directors can range from 200 hours per year to as much as 20 hours per week when companies are managing crises.^24^ Notably, although conflicts of commitment can and frequently do arise from roles that also create financial conflicts of interest and conflicting fiduciary duties, they can in principle also arise from outside activities involving entities whose interests are only marginally related or unrelated to those of the academic institution. In assessing whether a leader's outside activities pose conflict of commitment risks, oversight bodies should assess the cumulative demands of the leader's total portfolio of outside commitments and should recognize that time spent attending meetings or official events represents a lower bound of the time demands of external roles.

Fourth, there is a risk of *reputational harm* to the institution, either externally or internally, if leaders’ outside activities result in the public or the institution's own faculty, staff, or trainees questioning the leaders’ integrity or fidelity to the institutional mission. For example, a perfusionist at the Brigham suggested that knowing how much time the institution's president spent serving on corporate boards eroded staff morale and “left people shaking their heads.”^4^ Notably, reputational harms can occur whether or not an outside activity presents any of the risks noted previously. Some relationships, such as a university president serving on the board of a company that has been sanctioned for corporate malfeasance, may simply seem unsavory to external or internal observers.^20^

Fifth, particularly if leaders’ outside activities are seen as self‐serving, unnecessary, or poorly managed, there is a risk that they may erode the institution's *culture of conscience*. As described in the AAMC‐AAU's 2009 report: “Leading by personal example, officers and administrators should demonstrate to the academic community and to the public that responsible, accountable, and ethical behavior regarding financial conflicts of interest is an imperative, reflecting core institutional values.”^14^ Concerns about erosion of an institution's culture of conscience are closely related to concerns about internal reputational harm. However, whereas in the latter case, the risk is that leaders’ outside activities will be unpopular with internal stakeholders, the risk in the former is that leaders will set a poor example, thereby weakening trainees’, faculty members’, and staff members’ incentives to act ethically.

As with benefits, evidentiary support for some of the risks presented by leaders’ outside activities is limited and anecdotal. For example, it may not be clear how much, if any, reputational harm will be caused by a given external role. Thus, as noted previously, it is important that oversight bodies monitor for evidence of both foreseen and unanticipated risks and adjust policies and decisions accordingly.

### Management Strategies

When a leader's proposed outside activity is deemed to present risks to the institution, Step 4 of the decision‐making framework directs oversight bodies to assess whether those risks can be managed at reasonable cost to the institution. Navigating this step requires knowing what management strategies are available and then judging how well and at what cost some combination of those strategies can be expected to mitigate specific risks.

The AAMC‐AAU report enumerates several strategies for managing risks associated with leaders’ outside activities. Although not exhaustive, the list includes the most common strategies found in academic institutions’ ICOI management plans, such as^25^:

- *Disclosure* of the outside activity in multiple settings, including in publications and presentations, and to faculty, staff, and trainees under the leader's supervision;
- *Modification* of the outside activity, for instance by reducing time spent or compensation received;
- *Recusal* of the leader from institutional decisions that implicate the interests of the outside entity;
- *Monitoring* by independent and disinterested individuals or committees tasked with assessing bias in the leader's administrative decision making; and
- Designation of a *safe haven* (i.e., a nonconflicted senior faculty member or executive) with whom faculty, staff, and trainees can address concerns related to the leader's outside activity.

The feasibility and cost of these management strategies will likely vary across institutions and outside activities. Additionally, to the extent that different oversight bodies judge the magnitude of expected benefits differently, they will reach different conclusions about whether the costs of given management strategies are reasonable (i.e., proportional to the expected benefits). Nonetheless, it is possible to offer a general assessment of various management strategies applicability to different risks. Table 1 divides management strategies into those with lower administrative costs (disclosure and modification) and those with higher administrative costs (recusal, monitoring, and safe haven), and describes the rationale and limitations of employing each strategy in response to specific risks.

**Table 1:** ICOI Management Strategies’ Applicability to Specific Risks

	Management Strategies				
	Lower Administrative Cost		Higher Administrative Cost		
Risks	Disclosure	Modification	Recusal	Safe Haven	Monitoring
Financial COI	NA: Disclosure does not mitigate risk of bias.	Rational: Reducing or eliminating compensation for outside activity may mitigate risk of bias by reducing financial incentives.	Rationale: Preventing conflicted leader from directly influencing research, staffing, funding decisions related to outside interest may mitigate risk that important decisions are affected by bias.	Rationale: Faculty/staff given opportunity to voice concerns about conflicted leader's outside activity without fear of recrimination.	Rationale: Oversight provides a check against biased decision making (e.g., preferential treatment, misdirected resources).
		Limitations: Small financial incentives and relationships with outside entity may nonetheless affect decision making.	Limitations: Potentially difficult to identify and recuse conflicted leader from all decisions implicating outside interest. Administrative costs rise as conflicted leader is recused from more responsibilities.	Limitations: Faculty/staff may be uncomfortable using a safe haven. Administrative costs rise as more faculty/staff must be provided with safe haven.	Limitations: Monitoring committee will likely be limited in its ability to observe day‐to‐day decision making.
Conflicting fiduciary duties	NA: Disclosure does not resolve conflicting fiduciary duties.	NA: Limiting outside compensation or time commitment does not resolve conflicting fiduciary duties.	Rationale: Preventing conflicted leader from directly influencing research, staffing, funding decisions implicating competing fiduciary duties may mitigate risk that important decisions are affected by bias.	Rational: Faculty/staff given opportunity to voice concerns about conflicted leader's outside activity without fear of recrimination.	Rationale: Oversight provides a check against biased decision making (e.g., preferential treatment, misdirected resources).
			Limitations: Potentially difficult to identify and remove leader from all decisions implicating conflicting fiduciary duties. Administrative costs rise as conflicted leader is recused from more responsibilities.	Limitations: Faculty/staff may be uncomfortable using a safe haven. Administrative costs rise as more faculty/staff must be provided with safe haven.	Limitations: Monitoring committee will likely be limited in its ability to observe day‐to‐day decision making.
Conflict of commitment	NA: Disclosure does not address conflict of commitment.	Rationale: Reducing outside time commitment reduces risk that conflicted leader is unable to perform primary duties.	NA: Recusal does not address conflict of commitment. Conflict of commitment increases if recusal prevents conflicted leader from performing essential duties.	NA: Creation of a safe haven does not address conflict of commitment.	NA: Monitoring does not address conflict of commitment.
		Limitations: May be difficult to limit outside time commitment to the point that it poses no conflict of commitment.			
Reputational harm	Rationale: Demonstrating that institution has nothing to hide and welcomes scrutiny may improve public and internal perception of outside activity.	Rationale: Reducing outside compensation and/or time commitment may improve public and internal perception of outside activity.	Rationale: Demonstrating that leader and institution take risk of bias seriously may improve public and internal perception of outside activity.	Rationale: Demonstrating that leader and institution take risk of bias seriously may improve public and internal perception of outside activity.	Rationale: Demonstrating that leader and institution take risk of bias seriously may improve public and internal perception of outside activity.
	Limitations: Effects of transparency on public and internal perception are uncertain.	Limitations: Effects of activity modification on public and internal perception are uncertain.	Limitations: Effects of transparency on public and internal perception are uncertain.	Limitations: Effects of safe havens on public and internal perception are uncertain.	Limitations: Effects of monitoring on public and internal perception are uncertain.
Erosion of culture of conscience	Rationale: Shows that leaders comply with institutional rules, which may strengthen faculty, staff, and trainees’ incentives to act ethically.	Rationale: Reducing outside compensation and/or time commitment may strengthen faculty, staff, and trainees’ incentives to act ethically.	Rationale: Demonstrating that leader and institution take risk of bias seriously may strengthen faculty, staff, and trainees’ incentives to act ethically.	Rationale: Demonstrating that leader and institution take risk of bias seriously may strengthen faculty, staff, and trainees’ incentives to act ethically.	Rationale: Demonstrating that leader and institution take risk of bias seriously may strengthen faculty, staff, and trainees’ incentives to act ethically.
	Limitations: Effects of transparency on institutional culture are uncertain.	Limitations: Effects of activity modifications on institutional culture are uncertain.	Limitations: Effects of recusal on institutional culture are uncertain.	Limitations: Effects of safe havens on institutional culture are uncertain.	Limitations: Effects of monitoring on institutional culture are uncertain.

COI, conflicts of interest; NA, not applicable.

The analysis offers several lessons. First, most management strategies fail to address conflict of commitment. The only plausible way to address the problem of leaders’ overcommitting to outside activities is to limit time spent on those activities, which may not be possible in the case of an activity like board service that carries fixed and potentially expanding responsibilities. Second, strategies best suited to mitigating risks of biased decision making (recusal, safe havens, monitoring) carry potentially high administrative costs, which are likely to rise with the number and complexity of leaders’ outside activities and the scope of their responsibilities within their institutions. Although these may be effective strategies for mitigating the risk of biased decision making in principle, they may not be feasible or cost‐effective in practice. Third, strategies for preventing reputational harms and erosion of institutional culture rest on largely untested assumptions about how internal and external audiences are likely to perceive outside activities and the management strategies applied to them. Oversight bodies should, thus, be cautious in their assumptions about how effective these strategies are likely to be and, to the extent possible, monitor perceptions of their use. Although systematic assessment of external perceptions would likely prove challenging for most oversight bodies, consulting with faculty and staff representatives is feasible and could provide valuable insights into internal stakeholder perspectives. Overall, these considerations suggest that many ICOI may not be manageable at a reasonable cost to the institution.

## Applying the Framework

Thus far, we have described how an oversight body should assess the permissibility of academic leaders’ outside activities. We have yet to address the question of who should make up such a body. Effective real‐world application of this framework requires oversight bodies that are sufficiently independent and empowered to review and, if necessary, reject leaders’ proposed outside activities. Given that even senior faculty and staff within an academic institution may feel pressure to accommodate leaders’ wishes to pursue outside work, we endorse the recommendation included in the 2009 NAM report that an institution's board of trustees (or equivalent governing body) should establish a standing committee to review ICOI, including those related to leaders’ outside activities.^13^ Trustees bear responsibility for the institution at the highest level and have the authority to appoint and remove the highest ranking institutional official. Thus, unlike faculty and staff, they are not beholden to the goodwill of institutional leaders.

Although institutions may reasonably differ in their approaches to structuring board‐level ICOI committees, they should follow two key recommendations included in the 2009 NAM report. First, to encourage consideration of independent perspectives and guard against groupthink among trustees,  committees should include at least one member who is neither a member of the board nor an employee of the institution. Second, the ICOI committee should submit an annual report to the full board that both discusses how plans for managing or eliminating previous ICOIs were implemented and details any newly addressed ICOIs. To encourage accountability, suitably redacted versions of these reports should be made public. Publishing the reports might also help mitigate reputational risks associated with leaders’ outside activities by signaling the institution's commitment to maintaining a culture of conscience in which ICOI are taken seriously.

To demonstrate how our framework could guide an ICOI committee's assessment of a leader's proposed outside activity, we apply it to two cases. First, we consider a case drawn from Anderson and colleagues’^8^ study of academic leaders’ service on US health care company boards of directors, in which the chair of a department of medicine serves on the board of a pharmaceutical company (Box 1). Second, we consider a case adapted from the AAMC‐AAU guidance in which an oncology division chief serves on the scientific advisory board of a start‐up company, working to commercialize a technology developed in the chief's laboratory (Box 2).^14^ We argue that the activity proposed in the first case should not be allowed, whereas the activity proposed in the second case may be permissible.

### 1 Box 1. Case 1

**Background**: *The chair of a department of medicine has been invited to serve on the board of directors of a publicly traded pharmaceutical company whose product pipeline includes treatments for cardiovascular and metabolic diseases. As a director, the chair would be expected to attend quarterly board meetings as well as more frequent committee meetings and strategy sessions. Annual compensation for the role is $150,000*.

**Step 1: Does the activity involve an entity with closely related interests to those of the academic institution?**

- Yes.

**Step 2: Is the activity reasonably likely to yield substantial institutional or societal benefits?**

- The chair's board service could benefit the institution through increased knowledge, insights into emerging research directions and product development, ideas for new collaborative opportunities, identification of potential donors and other sources of financial support, or in other ways. The chair should be required to make a persuasive case for the reasonable likelihood of any such benefits before undertaking the role.
- The institutional conflict of interest (ICOI) committee should not allow the chair to accept the directorship and should not progress to the next step of the decision‐making framework if there is not a reasonable likelihood of the purported benefits being realized or the benefits are not deemed substantial.
- If the committee is persuaded that the role is likely to yield substantial benefits, it should ask whether those benefits can be realized at a lower level of involvement with the company or with the involvement of a less senior member of the academic institution.

**Step 3: Does the activity present ICOI or related risks?**

- The combination of factors in this case present risks of financial conflicts of interest and conflicting fiduciary duties. First, the directorship entails substantial financial compensation and fiduciary duties to the company's shareholders. Second, the company's interests intersect with the chair's oversight responsibilities for faculty members, staff, and trainees who may be working in areas related to the company's product portfolio. Thus, the chair's administrative decisions involving hiring, promotion, and other departmental matters may implicate the interests of the company and be unduly influenced by legal obligations and financial ties to the company.
- The case also presents conflict of commitment risks, which may be difficult to plan for given the need for board members to respond to unforeseen challenges.
- A department chair's external activities may not receive the same level of scrutiny as those of a health system chief executive officer or university president and, hence, may present lower risk of external reputational harm. However, there is a risk of internal reputational harm among department faculty, staff, and trainees who may view the directorship as a distraction or source of bias in the chair's decision making. Similarly, the role may present some risk to the departmental culture of conscience. If department members perceive the chair's outside role to be self‐serving or irresponsible, it may diminish their willingness to act ethically.

**Step 4: Can the concerns be managed at a reasonable cost to the institution?**

- Addressing risks of undue influence stemming from financial conflicts of interest and conflicting fiduciary duties would require some combination of recusal, safe havens, and external monitoring. Given the scope of their chair's oversight responsibilities, such measures would need to be extensive, likely carrying high administrative costs, and potentially precluding the chair from fulfilling core job functions. The chair could, in principle, decline compensation for the directorship, thereby mitigating concerns about financial conflicts of interest. However, even this exceptional step would not eliminate conflicting fiduciary duties.
- Conflict of commitment risks could, in principle, be addressed by modifying the external role to reduce time on board‐related activities. In practice, however, such modifications would likely be incompatible with the responsibilities of the directorship.
- It is possible that any reputational and culture of conscience risks could be mitigated through some combination of management strategies, which would demonstrate to external and internal audiences that the leader and institution have seriously considered potential harms and taken steps to address them, but the effectiveness of these measures is uncertain.

**Conclusion**

The directorship would present multiple risks that could be managed only at high administrative costs, if at all. Unless the chair is able to make a persuasive case that the institutional or societal benefits of the directorship would be so substantial as to outweigh these costs, which appears unlikely, the ICOI committee should not permit the outside activity. The chair and ICOI committee could consider alternative arrangements, such as the chair acting as a consultant to the company, which might present less risk to the institution but might also generate fewer institutional benefits.

### 2 Box 2. Case 2

**Background**: *An oncology division chief wants to serve on the scientific advisory board of a start‐up immuno‐oncology company that was spun out of the university to commercialize a novel immunotherapy technology developed in the chief's laboratory. The chief has declined compensation for the advisory board role but has an equity stake in the company as does the university. The advisory board is expected to convene quarterly*.

**Step 1: Does the activity involve an entity with closely related interests to those of the academic institution?**

- Yes.

**Step 2: Is the activity reasonably likely to yield substantial institutional or societal benefits?**

- As the inventor of the technology, the chief's input would likely be important to promote its successful development, thereby benefiting the institution by increasing the chances of a positive financial return on its equity stake in the company.
- The chief's role on the advisory board might generate societal benefits by helping to ensure that the technology is commercialized in a socially responsible manner, but as an advisory board member the chief would likely have limited input into the company's pricing and access strategies.
- As in case 1, the institutional conflict of interest (ICOI) committee should require the chief to make a persuasive case that the role is reasonably likely to produce these or other specific benefits before authorizing the role.

**Step 3: Does the activity present conflicts of interest or related risks?**

- Unlike case 1, the advisory board role would not entail fiduciary duties to the start‐up company. However, the chief's equity stake in the company presents a financial conflict of interest that could unduly influence his or her administrative decisions. Additionally, the chief would likely have a personal interest in seeing the company succeed, which may also pose a risk of undue influence.
- The conflict of commitment risks depend on the time demands on advisory board members. Assuming that advisory board meetings adhere to a schedule of quarterly meetings, the conflict of commitment risks involved in the external advisory board appear less severe than those of the directorship in the prior case.
- As in case 1, the chief's external activities are likely to receive less public scrutiny than those of university or health system executives. Thus, external reputational harm may not be a major concern. However, the advisory board role may still carry risks of internal reputational harms and risks to the division or broader institution's culture of conscience depending on how it is perceived by faculty, staff, and trainees.
- It is possible that the chief's service on the advisory board of a company developing technology from chief's laboratory would be seen as more closely connected to the mission of the institution and, hence, viewed more favorably than the directorship described in case 1. However, oversight bodies should be cautious in making assumptions about how leaders’ external activities will be received.

**Step 4: Can the concerns be managed at a reasonable cost to the institution?**

- As in case 1, mitigating the risk of undue influence stemming from the chief's financial and personal ties to the company would likely require some combination of recusal, creation of safe havens, and external monitoring to guard against undue influence. Similar to the prior case, these management strategies are likely to be extensive and to carry significant administrative costs given the chief's scope of responsibility.
- Assuming the time demands on advisory board members are fixed, there appears to be no way to mitigate conflict of commitment risks.
- Similar to case 1, risks of internal reputational harm and erosion of culture of conscience could be mitigated by the adoption of appropriate management strategies to address the risk of undue influence. Transparent communication both about the rationale and scope of the external role and the terms of the management plan may also mitigate reputational and culture of conscience risks.

**Conclusion**

The circumstances of this case differ from those of case 1 in several respects that should inform the ICOI committee's decision. First, although the risks of undue influence and the administrative costs of managing those risks are similar in the two cases, there appears to be a clearer prospect of institutional benefit in this case because of the institution's stake in the start‐up immuno‐oncology company. Second, because the division chief has unique insight into the company's underlying technology, it is unlikely that another member of the institution would be an equally effective member of the advisory board. Third, this case does not involve conflicting fiduciary duties. Fourth, this case involves less significant conflict of commitment than case 1. Fifth, but most speculatively, the nature of the chief's work with a company developing technology from the chief's laboratory may raise less risk of internal reputational harms.

Considering these factors, it would be reasonable for the ICOI committee to permit the chief to accept the outside role under an appropriate management plan. However, if the ICOI committee determined that the management strategies required to sufficiently mitigate the risk of undue influence were too costly or simply infeasible given institutional resources, it could reasonably prohibit the chief from taking the outside role. Alternative arrangements could involve the chief stepping down from a leadership position within the division.

## Conclusions

Academic leaders’ outside activities may contribute to institutional and societal benefits but also may expose institutions to substantial risks. Academic institutions have an ethical obligation to safeguard their ability to carry out their missions of advancing research, education, and patient care with high integrity. They also have pragmatic reasons to avoid negative media coverage and public skepticism about their leaders’ priorities. Applying the practicable, flexible, yet rigorous approach we describe to identifying and weighing benefits and risks can help shift scrutiny of leaders’ outside activities to where it belongs: in institutional governance structures and not the front page of newspapers.

## Conflict of Interest Disclosures

S.J. is a paid member of a data and safety monitoring committee for CSL Behring. B.L. reports the following: honoraria from Blue Cross Blue Shield; member, Medical Advisory Panel, which recommends whether scientific evidence shows that a technology provides a net health benefit; honoraria from Vivli, which advocates for data sharing by clinical trials; member, External Advisory Committee; honoraria from Takeda Pharmaceuticals, Ethics Advisory Committee, which recommends guiding ethical principles on such topics as data sharing, gene and cell therapies, artificial intelligence, and digital and social media engagement; unpaid member of Institutional Conflict of Interest Committee, MD Anderson Cancer Center; and unpaid chair, chair, External Advisory Board, Multi Regional Clinical Trials Center, Harvard University, which aims to improve the quality, transparency, and ethics of multiregional clinical trials.

## Funding/Support

This work was funded by a grant to the University of Pennsylvania from the National Center for Advancing Translational Sciences of the National Institutes of Health (5UL1TR001878). The sponsor had no input into this research or publication.
